# Breaking New Ground: Incentivizing Innovative Caregiving Programs

**DOI:** 10.1093/geroni/igab046.1185

**Published:** 2021-12-17

**Authors:** C Grace Whiting, Theresa Harvath

## Abstract

Caring for someone with chronic illness is a demanding job, and as a result 46% of caregivers caring for adults with chronic illnesses report a significant level of burden (AARP and National Alliance of Caregiving, 2015). Recent reviews note a prevalence rate of 31.2% for depression (Collins & Kishita, 2019) and 32.1% for anxiety (Kaddour & Kishita, 2020). In addition, most caregivers also report high levels of negative emotions including frustration, guilt, and a sense of hopelessness regarding the future (Schulz & Eden, 2016). This symposium will focus on innovative programming to address caregiver needs and concerns. The first presenter will set the foundation as she explores her caregiver journey and the issues she experienced as a caregiver. Using her personal experience, this healthcare professional will explore her interactions with the medical system as a caregiver, including the unique issues experienced during the pandemic. The second presenter will examine why are caregiver program needed, what benefits can be expected, and what is considered best practices when addressing the unmet needs of family caregivers in caregiver programs. The third and fourth presenters will discuss two exemplar caregiving programs - Caregiver Clinic at the Memorial Sloan Kettering Cancer Center and the Caregiver Initiative from the Rush University Medical Center. They will describe the program, the process of creation, funding, barriers experienced, and working solutions. Pertinent data regarding the integration of the programs within the medical systems, the programs scope, and the effects of the pandemic on the programs will be shared.

